# Postage on Scientific Journals

**Published:** 1850-02

**Authors:** 


### POSTAGE ON SCIENTIFIC JOURNALS.

Of all the abominations of the present system of American postage, none seems to us more perfectly wrong than the tax imposed upon scientific periodiculs. The very erroneous doctrine, that the Post Office Department must pay its own expenses, which has always governed the postal arrangements of the general government, is the root of the evil. The immediate error of action, springing from this doctrine, is found in the distribution of the tariff on mail matters, by which newspapers are carried at a loss to the department, and letters, literary magazines, and scientific publications are required not only to pay for their carriage, but to make up the losses sustained from the diminutive rates charged on newspapers. What shadow of reason is there for making scientific periodicals pay exorbitant postage on their exchanges, that is not equally imperative on the newspaper press, which pays no postage on newspapaper exchanges?

A great and beneficent reform in the entire machinery of the Post Office Department is in progress, and it behooves all those interests that feel the wrongs of the present system, to speak out. The principles on which the General Post Office should be made to operate, is one that is vital to the welfare of the people: it is, that intelligence shall be distributed to all parts of the country alike; and on the same terms, so that a beggar may be placed, by the action of the government, on a footing of equality with the millionaire in the enjoyment of intelligence. This is the principle on which the British Post Office has been working for nearly ten years, and the results have been of the most beneficent character. That Rowland Hill's system would work as effectively in this country may be proved by a reference to some of the prominent facts in that system, and the American plan of operations. The management of the British Post Office costs $6,712,368—that of this country costs $4,346,850. The cost of transportation in Great Britain is $2,229,-763, while in the United States, notwithstanding "the immense distances traversed by our mails," the same service costs $2,448,756, only $210,983 more."*

Our cotemporary of the Southern Medical and Surgical Journal urges the scientific journals of the United States to efficient action in effecting a reform in the postage tariff of exchange journals. We should not rest at that: it is the bounden duty of Congress, not only to effect that reform, but to encourage the distribution of scientific intelligence among the people. To say nothing of other beneficial effects of the general distribution of scientific truth among the people, it will be admitted that when the great mass of the people of this country is thoroughly imbued with a knowledge of geology, scientific agriculture, and the principles of public hygeine, health, prosperity, peace and virtue will be the fruits of these gifts. These are a part of the objects for which the scientific journals of this country labor, and it is upon such labors that our wise statesmen lay their heaviest burdens. The increased prosperity of the people, arising from accurate knowledge of public hygeine, would more than compensate the general government for carrying scientific journals in the mails gratis.

We join the Southern Medical and Surgical Journal in urging on the editors and proprietors of all scientific journals in the country, the necessity of making an immediate effort in this special postage reform, of which we have spoken. Each Journal should at once address itself to the chairman of the Post Office Committee, and endeavor to have the feature, referred to, introduced into the Post Office bill.

---

### THE MEDICAL JOURNAL FOR MARCH.

The numerous readers of this Journal will be gratified by the information we give them, that Prof. Bartlett is preparing an article for our March No. His paper is on a subject that is exceedingly interesting in itself; what it will be in his hands, it is scarcely necessary for us to say.

---

* **Hunt's Merchant's Magazine.**

